# Education-related differences in physical performance after age 60: a cross-sectional study assessing variation by age, gender and occupation

**DOI:** 10.1186/1471-2458-13-641

**Published:** 2013-07-10

**Authors:** Anna-Karin Welmer, Ingemar Kåreholt, Elisabeth Rydwik, Sara Angleman, Hui-Xin Wang

**Affiliations:** 1 Aging Research Center (ARC), Department of Neurobiology, Care Sciences and Society, Karolinska Institutet, Stockholm University, 16, S-113 30 Stockholm, Sweden; 2 Karolinska University Hospital, Stockholm, Sweden; 3 Institute for Gerontology, School of Health Sciences, Jönköping University, Jönköping, Sweden; 4 Research and Development Unit, Jakobsbergs Hospital, Järfälla, Sweden

**Keywords:** Educational status, Aging, Chronic diseases, Muscle strength, Walking, Postural balance

## Abstract

**Background:**

Having a low level of education has been associated with worse physical performance. However, it is unclear whether this association varies by age, gender or the occupational categories of manual and non-manual work. This study examined whether there are education-related differences across four dimensions of physical performance by age, gender or occupational class and to what extent chronic diseases and lifestyle-related factors may explain such differences.

**Methods:**

Participants were a random sample of 3212 people, 60 years and older, both living in their own homes and in institutions, from the Swedish National Study on Aging and Care, in Kungsholmen, Stockholm. Trained nurses assessed physical performance in grip strength, walking speed, balance and chair stands, and gathered data on education, occupation and lifestyle-related factors, such as physical exercise, body mass index, smoking and alcohol consumption. Diagnoses of chronic diseases were made by the examining physician.

**Results:**

Censored normal regression analyses showed that persons with university education had better grip strength, balance, chair stand time and walking speed than people with elementary school education. The differences in balance and walking speed remained statistically significant (p < 0.05) after adjustment for chronic diseases and lifestyle. However, age-stratified analyses revealed that the differences were no longer statistically significant in advanced age (80+ years). Gender-stratified analyses revealed that women with university education had significantly better grip strength, balance and walking speed compared to women with elementary school education and men with university education had significantly better chair stands and walking speed compared to men with elementary school education in multivariate adjusted models. Further analyses stratified by gender and occupational class suggested that the education-related difference in grip strength was only evident among female manual workers, while the difference in balance and walking speed was only evident among female and male non-manual workers, respectively.

**Conclusions:**

Higher education was associated with better lower extremity performance in people aged 60 to 80, but not in advanced age (80+ years). Our results indicate that higher education is associated with better grip strength among female manual workers and with better balance and walking speed among female and male non-manual workers, respectively.

## Background

Population aging leads to an increase in the number of people with disability, thus placing growing demands on health care and social services. Strong epidemiological evidence suggests that physical performance measures are reliable markers of current health and independent predictors of disability, cognitive decline and mortality among older adults [1,2]. It has been suggested that physical limitations are more common among older adults with low education and low socioeconomic position [3-7]. Less is known however about the underlying mechanisms and pathways associated with education-related differences in different dimensions of physical performance.

It has been suggested that exposure to psychosocial risk factors among persons with lower education or the impact of these factors on health may be greater among people in middle age and early old age compared to the oldest-old [8]. However, earlier reports on age variations in social inequalities in health are contradictory. Some studies show greater differences with age [9,10], while others suggest that the differences in health are most pronounced in midlife and early old age and then converge in old age [8,11,12]. Female gender has been associated with physical limitations [13,14], and the gender differences have been suggested to further increase with higher levels of education [14]. Furthermore, health inequalities may also have different explanations in men and women [15,16]. For example, men may be more exposed or vulnerable to work-related risk factors in terms of musculoskeletal problems [17].

Unfavourable lifestyle-related factors such as physical inactivity, obesity, smoking and heavy alcohol consumption may be more common among people in socially disadvantaged groups [7,18-20]. It is therefore possible that the education-related differences in physical performance may be influenced by these factors. Indeed several studies have found that adjusting for lifestyle-related factors reduces differences in functional limitations between socioeconomic groups [6,7,21]. However, few studies employed objective measurements of physical performance—as opposed to self-reported functional limitations [3,5,6]. One study found that obesity and smoking accounted for some of the education-related differences in mobility among older adults [6]. Another study reported that adjustment for smoking and physical activity made only small changes to the associations between education and walking speed [5]. Some studies have found that chronic diseases explain much of the socioeconomic inequalities in functional limitations [5,6,22], while others suggest that adjustment for chronic diseases only slightly attenuates the differences between the social groups [5,6,22]. Finally, manual workers have been found to have a higher risk of developing osteoarthritis due to the strenuousness of their work [23]. Thus, manual work may contribute to physical limitations and education-related differences among older adults [6,24]. It is however not known whether education is differently associated with physical performance in the occupational categories of manual and non-manual work.

On the basis of previous research, we hypothesize that (1) higher education is associated with better physical performance, in which the association may vary by age and gender, and (2) the occupational categories of manual and non-manual work may modify the associations differently for men and women. In this population-based study of people aged 60 years and older, we seek to test these hypotheses by examining 1) the education-related differences in four dimensions of physical performance, i.e. grip strength, balance, chair stands and walking speed, 2) whether the possible differences vary by age, gender or occupational class and 3) to what extent chronic diseases and lifestyle-related factors (physical exercise, body mass index, smoking and alcohol consumption) may explain such differences.

## Methods

### Study sample

This study used data from one of four subprojects included in the Swedish National Study on Aging and Care (SNAC), the Kungsholmen population study (SNAC-K) [25]. The population was stratified by age and then a random sample was selected from each selected age cohort. Eleven age cohorts were chosen with different age intervals, which was six years in the younger cohorts (60–78 years) and three years in the older cohorts (81- 99+ years). The baseline data collection was conducted from March 2001-June 2004. A total of 5111 persons were initially selected to be invited for participation, of these 4590 were alive and eligible to participate (200 dead, 262 not able to be contacted, 4 deaf, 23 did not speak Swedish, and 32 had moved). Of those eligible, 3363 (73.3%) participated at the baseline examination. Thus, the persons studied at the baseline visit of SNAC-K consisted of 3363 persons aged 60 years and older, both living in their own homes and in institutions, in Kungsholmen, Stockholm. SNAC-K was approved by the Ethics Committee at Karolinska Institutet and by the Regional Ethical Review Board in Stockholm. Informed written consent was obtained directly from participants. If the person was cognitively impaired, a proxy was asked for consent (usually a close family member).

Subjects with missing values in the lower extremity physical performance tests due to the inability to walk without personal support (N = 117) received the worst possible score, i.e. 0 seconds balance time, 0 chair stands/minute or a walking speed of 0 m/s. For grip strength, subjects with physical or cognitive problems (N = 12) or those who refused to participate (N = 7) were assigned a score = 0. For the lower extremity tests, data was missing in one of the tests for 295 subjects, in two of the tests for 108 subjects, and in all three tests for 40 subjects. For subjects with missing values in one or two of the three lower extremity tests, values were imputed by using age, gender and the other lower extremity-related tests as predictors. For subjects with missing values in grip strength (N = 746), values were imputed using age, gender, the lower extremity-related tests, death (yes/no), time to death, location of the interview (the test centre vs. own homes), limitations in any activity of daily living (yes/no) (ADL; i.e. dressing, hygiene, mobility, bathing/showering, eating) and a question about whether the participant could open cans with screw caps (without difficulties/yes, with difficulties/no) as predictors. In total, grip test data was available for 3210 persons (including 593 with imputed score); lower extremity data was available for 3323 persons (including 136 with imputed score for chair stands, 144 with imputed score for walking speed, and 194 with imputed score for balance score) [13]. We excluded 153 subjects for whom we could not calculate imputed scores for grip strength, and 40 subjects for the lower extremity tests due to lack of data in the variables used for imputation. Thirty-two subjects had missing data in education, and 120 had missing data in occupational class, leaving 3155 people (mean age ± SD, 73.9 ± 10.9, 63.3% women) available for the current analysis on grip strength, and 3212 (mean age ± SD, 74.2 ± 11.0, 63.8% women) available with data on lower extremity performance. The excluded subjects (n = 208 for grip strength and n = 151 for lower extremity performance) were significantly older, more often women and had worse ADL capacity, compared to the included subjects (p < 0.05). The subjects with imputed values (n = 553 for grip strength, n = 189 for balance, n = 103 for chair stands and n = 136 for walking speed) had significantly worse physical performance on all tests, compared to those without imputed values (p < 0.05).

### Data collection

SNAC-K data was collected at our research center via interviews, clinical examinations and testing by trained staff. For those who agreed to participate but were unable or unwilling to come to the research centre, home visits were conducted (n = 717). Those who received home visits were significantly older, more often women and had lower levels of education, worse ADL capacity and physical performance, compared to those who were assessed at the research centre (p < 001). The assessment of physical performance was performed by trained nurses and included tests of grip strength, chair stands, walking speed and balance. The lower extremity tests were derived from the performance battery used in the Established Populations for the Epidemiologic Study of the Elderly (EPESE) [26].

*Grip strength* was assessed with the Grippit [27], which is an electronic device measuring maximum grip strength in Newtons. The participant squeezes a handle with maximum force, once with each hand. The best overall value was used in the analysis. Grip strength was not assessed at home visits because it was infeasible to transport the measurement equipment to the homes of all subjects.

The measure of *balance* was based on the length of time the subject was able to stand on one leg with eyes open [28]. The test was performed twice on each leg, and the best overall value was used in the analysis. Subjects were timed up to a maximum of 60 seconds for each trial.

*Chair stands* were assessed by asking the participants to fold their arms across their chests and to stand up from a sitting position once. If they were able to successfully rise from the chair, they were asked to stand up and sit down five times as quickly as possible. The time required was measured in seconds [29]. In the analyses, the results are presented as chair stands per minute.

*Walking speed* was assessed by asking the participants to walk 2.4 or 6 meters at a self-selected speed [29]. The length of the walk was determined by asking the participants how fast they normally walk. Subjects who rated themselves as fast or normal walkers did the longer walk and slow or very slow self-rated walkers did the shorter walk. At home visits, the shorter walk was always conducted due to space restrictions. For the analyses, the walking speed reflects the time from whichever test was performed, presented in meters per second.

Information on *the highest level of formal education* (elementary school, high school or university) was assessed directly from the subject or from an informant.

Medical diagnoses were made by the examining physicians based on the clinical examination, self-reported medical history and laboratory data. *Chronic diseases* were evaluated by counting the number of prevalent chronic diseases/conditions at the time of the study visit. A disease/condition was classified as chronic if it was prolonged in duration and if one or more of the following characteristics were present: 1) leaving residual disability or worsening of quality of life; and/or 2) requiring a long period of care or treatment/rehabilitation [30]. Each subject was classified as affected by a disease when diagnosed by the examining physician. The International Classification of Diseases (ICD 10) was used to classify diseases. Chronic diseases were analysed as an indicator variable (maximum 14 diseases).

*Leisure-time physical exercise* was measured by two survey questions: ‘How often did you exercise with light intensity (e.g. walks on the sidewalk or paved surfaces, in parks, in forests, short bike rides, light gymnastics, golf) in the last 12 months?’, and 2) ‘How often did you exercise more intensively (e.g. brisk walking, jogging, heavy gardening, long bike rides, intense gymnastics, skating, skiing, swimming, ball games or similar activities) in the last 12 months?’. Based on research recommendations, the subjects were categorized in three groups according to the levels of the activities (from low to high): 1) inadequate: ≤2-3 times per month in light and/or intensive activity; 2) health-enhancing: light exercise several times per week or every day; and 3) fitness-enhancing: moderate/intense exercise several times per week or every day [31].

*Body mass index (BMI)* was calculated by dividing weight in kilograms by height in meters squared. It was categorized as underweight (<20), normal weight (20–24.9, used as the reference category in the analysis), overweight (25–29.9) and obese (≥30 kg/m^2^) [32].

*Smoking status* was categorized into never, former, and current smoking. *Alcohol consumption* was categorized into no or occasional, light-to-moderate (1–14 drinks per week for men or 1–7 drinks per week for women) or heavy (≥15 drinks per week for men or ≥8 drinks per week for women) [33]. A standard drink (approximately 12–14 g pure ethanol) is equal to 330 ml beer, 150 ml wine or 40 ml spirit.

Data on age, gender and the longest held occupation were derived from the nurse interview. *Occupational class* during the longest held occupation was based on types of occupation and was categorized into two groups (according to classifications determined by Statistics, Sweden): 1) manual workers: no trained skill, goods-producing; no trained skill, service-producing; trained skill, goods-producing; trained skill, service-producing; 2) non-manual workers: junior office worker, less than two years; education after elementary school; junior office worker, two but not three years after elementary school; office worker, three but not six years after elementary school; senior office worker, at least six years after elementary school; entrepreneurs; academic professions. If the subjects could not give information about time periods but gave information on type of work, the most recent work before retirement was used in the analyses.

### Data analysis

Quantile regression analyses were used to calculate age-adjusted median scores and interquartile ranges (between the value corresponding to the 25% and 75% percentiles) of physical performance according to characteristics of the study population, stratified by age group (<80 vs. ≥80 years) and gender. For the calculation of the age-adjusted median scores and percentiles, three effect coded age variables were created; one for the total sample which was used when stratifying by gender, one for people <80 years and one for people ≥80 years. Education-related differences in physical performance and lifestyle were calculated using one-way ANOVA, t-test or Chi-square test.

Censored normal regression analyses were conducted to examine the associations between education and each of the physical performance tests. We used censored normal regression analyses because several values of the dependent variables were censored at zero or at the maximum score [34]. The censored values were treated as if they have the minimum score or less or the maximum score or more. All outcome variables were log-transformed, which makes it a multiplicative model, and adjusted with a constant to approximate normal distributions. The results were presented as percentage difference compared to a reference group, which was calculated as follows: (the relative difference, i.e. the exponential coefficient −1) * 100.

We performed censored normal regression models with education as the independent variable, and each of the physical performance tests as dependent variables. In the first model we adjusted for age and gender. In other models, additional adjustments were made separately for the number of chronic diseases, and each of the lifestyle-related factors. In a final model, we adjusted for age, gender, chronic diseases and all lifestyle-related factors simultaneously. We created indicator variables to replace missing values for those variables that had missing values (chronic diseases (n = 9), BMI (n = 227), smoking (n = 33) and alcohol consumption (n = 30), total n = 259). Differences between the statistical models were calculated using t-tests.

Stratified analyses by age, gender and occupational class were performed to investigate whether these associations are different in people aged ≥80 years and people aged <80 years, in men and women and in manual and non-manual workers. The cut-off of 80 years represents a population-based definition of the oldest people since about 50% of all people in Sweden die before 80 years of age [35]. Statistical interactions were tested by simultaneously including the independent variables and their cross-product variables in the same model. The analyses were performed using STATA 11 software.

## Results

In examining age-adjusted median levels of each of the four different objective tests of physical performance, lower physical performance was found in older participants, in women, in manual workers, in people with lower levels of education, with two or more chronic diseases, with lower levels of physical exercise and with no or heavy alcohol consumption, as well as in those who were either underweight or obese (Tables 1 and 2).

**Table 1:** Characteristics of the 3212 participants and their age-adjusted median scores (Interquartile ranges) of physical performance by gender from quantile regression models

	**<80 years (n = 2211)**					**≥80 years (n = 1001)**				
	***n***	Grip strength* (Newton)	**Balance score (0–60 sec)**	**Chair stands (/minute)**	**Walking speed (m/s)**	***n***	**Grip strength† (Newton)**	**Balance score (0–60 sec)**	**Chair stands (/minute)**	**Walking speed (m/s)**
Gender										
Men	*904*	365 (313–418)	36 (16–49)	26 (19–33)	1.1 (1.0-1.4)	*260*	271 (226–320)	1 (0–6)	7 (0–15)	0.7 (0.4-1.0)
Women	*1307*	202 (163–236)	36 (13–49)	23 (17–29)	1.1 (1.0-1.4)	*741*	149 (124–171)	1 (0–4)	7 (0–12)	0.4 (0.3-0.8)
Education										
Elementary school	*239*	221 (180–335)	30 (10–49)	21 (15–29)	1.1 (0.7-1.4)	*314*	162 (128–201)	1 (0–4)	6 (0–11)	0.4 (0.3-0.7)
High school	*1059*	231 (183–327)	34 (13–49)	23 (17–29)	1.1 (0.9-1.4)	*526*	160 (132–190)	1 (0–4)	6 (0–13)	0.6 (0.3-0.8)
University	*913*	263 (204–358)	37 (20–49)	27 (20–33)	1.3 (1.1-1.4)	*161*	201 (150–262)	1 (0–5)	9 (0–18)	0.7 (0.4-0.8)
Occupational class										
Manual workers	*395*	232 (186–325)	34 (14–49)	22 (14–27)	1.1 (0.8-1.4)	*373*	159 (129–191)	1 (0–4)	7 (0–10)	0.4 (0.2-0.7)
Non-manual workers	*1816*	247 (191–344)	36 (16–49)	24 (18–30)	1.1 (1.0-1.4)	*628*	167 (136–224)	1 (0–4)	7 (0–13)	0.6 (0.3-0.8)
Chronic diseases										
0-1 diseases	*1272*	251 (200–348)	39 (18–49)	26 (20–33)	1.3 (1.0-1.4)	*284*	169 (138–223)	1 (0–7)	10 (0–17)	0.7 (0.4-1.0)
2+ diseases	*935*	234 (179–330)	31 (11–49)	22 (15–28)	1.1 (0.8-1.4)	*712*	162 (131–200)	1 (0–4)	6 (0–11)	0.4 (0.2-0.7)
Physical exercise										
Inactive	*490*	237 (181–331)	27 (10–49)	20 (8–26)	0.9 (0.7-1.2)	*554*	161 (129–196)	0 (0–2)	0 (0–5)	0.4 (0.2-0.6)
Health-enhancing	*1129*	237 (189–331)	38 (16–49)	24 (18–30)	1.1 (1.0-1.4)	*381*	168 (137–216)	4 (0–7)	16 (0–14)	0.7 (0.5-0.9)
Fitness-enhancing	*592*	260 (200–366)	38 (21–49)	27 (21–34)	1.3 (1.0-1.4)	*66*	178 (142–247)	5 (0–10)	20 (0–14)	0.9 (0.7-1.1)
Body mass index (kg/m2)										
<20	*76*	222 (199–278)	37 (11–49)	21 (3–29)	1.1 (0.8-1.4)	*118*	156 (135–193)	1 (0–3)	7 (0–12)	0.4 (0.3-0.7)
20-24.99	*843*	233 (188–317)	37 (17–49)	25 (20–33)	1.3 (1.0-1.4)	*374*	169 (133–212)	1 (0–6)	7 (0–20)	0.7 (0.4-0.9)
25-29.99	*940*	259 (194–368)	37 (15–49)	24 (18–29)	1.1 (1.0-1.4)	*246*	170 (134–224)	1 (0–7)	9 (0–18)	0.7 (0.4-0.9)
≥30	*321*	245 (182–340)	28 (11–49)	22 (16–27)	1.1 (0.8-1.4)	*68*	159 (136–199)	1 (0–4)	8 (0–17)	0.7 (0.5-0.8)
Smoking										
Never	*912*	230 (183–319)	36 (16–49)	24 (19–30)	1.1 (1.0-1.4)	*590*	157 (129–186)	1 (0–4)	7 (0–13)	0.5 (0.3-0.8)
Former	*909*	263 (200–360)	36 (16–49)	24 (18–30)	1.1 (1.0-1.4)	*315*	180 (147–262)	1 (0–4)	7 (0–15)	0.6 (0.4-0.8)
Current	*377*	240 (196–338)	36 (16–49)	22 (17–28)	1.1 (0.8-1.4)	*76*	175 (137–237)	1 (0–5)	7 (0–13)	0.6 (0.4-0.8)
Alcohol consumption										
No or occasional	*565*	221 (172–293)	30 (11–49)	21 (14–27)	1.1 (0.8-1.4)	*600*	161 (130–188)	1 (0–3)	4 (0–12)	0.4 (0.2-0.7)
Light to moderate	*1217*	272 (201–368)	37 (18–49)	25 (20–33)	1.1 (1.0-1.4)	*299*	184 (139–272)	1 (0–7)	12 (0–17)	0.7 (0.4-1.0)
Heavy	*419*	225 (185–284)	37 (15–49)	24 (18–29)	1.1 (1.0-1.4)	*82*	160 (133–191)	1 (0–6)	12 (0–18)	0.7 (0.5-0.8)

Grip strength in people <80 years: *=2209 participants; grip strength in people ≥80 years †=946 participants. Imputed values were used for grip strength (n = 92 for people <80 years and n = 461 for people 80+ years), balance (n = 59 for people <80 years and n = 130 for people 80+ years), chair stands (n = 41 for people <80 years and n = 62 for people 80+ years) and walking speed (n = 43 for people <80 years and n = 93 for people 80+ years). Numbers of missing values were 9 for chronic diseases (4 among people <80 years and 5 among people 80+ years), 227 for BMI (32 among people <80 years and 195 among people 80+ years), 33 for smoking (13 among people <80 years and 20 among people 80+ years) and 30 for alcohol consumption (10 among people <80 years and 20 among people 80+ years).

**Table 2:** Characteristics of the 3212 participants and their age-adjusted median scores (Interquartile ranges) of physical performance by gender from quantile regression models

	**Men (n = 1164)**					**Women (n = 2048)**				
	***n***	Grip strength* (Newton)	**Balance score (0–60 sec)**	**Chair stands (/minute)**	**Walking speed (m/s)**	***n***	**Grip strength† (Newton)**	**Balance score (0–60 sec)**	**Chair stands (/minute)**	**Walking speed (m/s)**
Age groups										
60-66	*566*	397 (343–450)	60 (29–60)	30 (21–38)	1.2 (1.2-1.5)	*728*	225 (184–261)	60 (23–60)	27 (21–33)	1.2 (1.2-1.5)
72-78	*338*	331 (279–383)	14 (5–47)	21 (17–27)	1.2 (0.8-1.2)	*579*	179 (142–210)	11 (4–34)	20 (12–25)	1.0 (0.8-1.2)
81-87	*179*	276 (227–329)	3 (0–10)	17 (0–23)	0.8 (0.5-1.2)	*411*	153 (125–177)	3 (0–7)	9 (0–20)	0.6 (0.4-1.0)
90+	*81*	266 (224–308)	0 (0–3)	0 (0–9)	0.5 (0.3-0.8)	*330*	145 (122–166)	0 (0–0)	0 (0–3)	0.3 (0.0-0.5)
Education										
Elementary school	*164*	316 (266–364)	19 (8–30)	16 (9–22)	0.9 (0.7-1.2)	*389*	172 (135–196)	18 (6–25)	13 (8–17)	0.8 (0.5-1.0)
High school	*487*	318 (267–368)	19 (8–30)	16 (9–24)	0.9 (0.7-1.2)	*1098*	173 (143–201)	18 (6–25)	13 (8–20)	0.8 (0.6-1.1)
University	*513*	318 (270–366)	21 (11–30)	18 (11–26)	1.0 (0.7-1.2)	*561*	185 (155–214)	19 (9–25)	16 (10–23)	1.0 (0.8-1.1)
Occupational class										
Manual workers	*204*	317 (265–363)	19 (7–30)	15 (8–20)	0.9 (0.6-1.2)	*564*	176 (144–202)	18 (6–25)	11 (8–19)	0.8 (0.5-1.0)
Non-manual workers	*960*	318 (269–367)	19 (8–30)	18 (10–25)	0.9 (0.8-1.2)	*1484*	176 (142–204)	18 (7–25)	15 (8–21)	0.8 (0.7-1.0)
Chronic diseases										
0-1 diseases	*630*	330 (273–374)	22 (10–34)	18 (12–26)	1.0 (0.8-1.2)	*926*	181 (153–208)	19 (7–25)	16 (9–23)	0.9 (0.7-1.1)
2+ diseases	*531*	313 (266–360)	17 (8–24)	15 (9–23)	0.8 (0.6-1.2)	*1116*	172 (137–201)	17 (7–25)	12 (8–19)	0.8 (0.5-1.0)
Physical exercise										
Inactive	*334*	308 (261–350)	16 (7–30)	13 (6–19)	0.7 (0.5-1.0)	*710*	171 (139–199)	16 (6–23)	8 (7–14)	0.6 (0.4-0.8)
Health-enhancing	*536*	320 (271–378)	22 (9–30)	18 (13–24)	1.0 (0.9-1.2)	*974*	179 (143–206)	20 (6–26)	18 (10–22)	0.9 (0.8-1.1)
Fitness-enhancing	*294*	331 (278–377)	22 (12–30)	22 (15–26)	1.1 (0.9-1.2)	*364*	182 (155–211)	20 (11–26)	21 (15–26)	1.0 (0.8-1.1)
Body mass index (kg/m2)										
<20	*48*	309 (268–346)	20 (9–30)	16 (9–19)	0.8 (0.6-1.0)	*146*	177 (143–199)	19 (15–24)	14 (9–21)	0.8 (0.6-1.0)
20-24.99	*401*	313 (267–359)	20 (9–30)	18 (9–26)	1.0 (0.8-1.2)	*816*	177 (145–205)	19 (8–26)	16 (9–25)	0.9 (0.7-1.1)
25-29.99	*534*	326 (276–374)	20 (9–30)	18 (11–26)	1.0 (0.8-1.2)	*652*	174 (145–203)	19 (7–26)	14 (9–23)	0.8 (0.7-1.1)
≥30	*154*	321 (260–379)	15 (3–30)	15 (8–22)	0.9 (0.6-1.2)	*234*	170 (135–202)	15 (4–26)	14 (7–21)	0.8 (0.5-1.0)
Smoking										
Never	*406*	319 (269–368)	19 (9–30)	17 (10–26)	1.0 (0.7-1.2)	*1096*	174 (141–202)	18 (7–25)	15 (9–21)	0.8 (0.6-1.1)
Former	*577*	317 (266–367)	19 (9–30)	17 (10–23)	0.9 (0.7-1.2)	*647*	177 (145–204)	18 (7–25)	12 (8–21)	0.8 (0.6-1.1)
Current	*174*	317 (274–358)	19 (7–30)	17 (10–22)	0.9 (0.7-1.2)	*279*	178 (150–206)	18 (4–25)	13 (8–21)	0.8 (0.6-1.1)
Alcohol consumption										
No or occasional	*277*	304 (260–361)	19 (9–30)	16 (9–21)	0.9 (0.6-1.2)	*888*	174 (138–201)	16 (7–25)	12 (8–19)	0.7 (0.5-1.0)
Light to moderate	*756*	323 (276–369)	20 (9–30)	18 (10–26)	1.0 (0.8-1.2)	*760*	175 (145–204)	20 (7–25)	17 (9–23)	0.9 (0.7-1.1)
Heavy	*122*	312 (279–360)	19 (4–30)	16 (9–22)	0.9 (0.8-1.2)	*379*	181 (154–209)	20 (7–25)	17 (9–23)	0.9 (0.7-1.1)

Grip strength in men *=1158 participants; grip strength in women †=1997 participants. Imputed values were used for grip strength (n = 138 for men and n = 415 for women), balance (n = 54 for men and n = 135 for women), chair stands (n = 32 for men and n = 71 for women) and walking speed (n = 37 for men and n = 99 for women). Numbers of missing values were 9 for chronic diseases (3 among men and 6 among women), 227 for BMI (27 among men and 200 among women), 33 for smoking (7 among men and 26 among women) and 30 for alcohol consumption (9 among men and 21 among women).

Persons with university education had 23% better balance, 26% better chair stands, 18% faster walking speed and 4% better grip strength compared to persons with elementary education, when adjusting for age and gender (Table 3). In the fully adjusted model controlling for age, gender, chronic diseases and lifestyle-related variables, the differences were attenuated but remained statistically significant for balance and walking speed (Table 3). In addition, we observed a gradient difference between levels of education and walking speed. Higher levels of education were associated with faster walking speeds. People who had a university education had 18% faster walking speed, and those who had a high school education had 7% faster walking speed, as compared to those who only had an elementary school education. This association persisted even after adjusting for a number of different confounders, although the statistical significance for high school education no longer existed in the fully adjusted model (Table 3). However, the differences between results from the different models were not statistically significant for any of the outcomes.

**Table 3:** Adjusted percentage difference in grip strength, balance, chair stands and walking speed, for subjects with high school or university education compared to subjects with elementary school education from censored normal regression models

**Adjusted for**	**Grip strength**	**Balance**	**Chair stands**	**Walking speed**
	**(n = 3155)**	**(n = 3212)**	**(n = 3212)**	**(n = 3212)**
Age and gender				
High school	1.5 (−1.6-4.7)	6.7 (−0.4-14.4)	9.7 (−2.2-23.0)	7.3 (3.4-11.4) ***
University	4.1 (0.6-7.7)*	22.8 (14.1-32.3)***	26.3 (12.3-42.1) ***	17.8 (13.1-22.7) ***
+ Chronic diseases				
High school	1.2 (−1.9-4.4)	6.4 (−0.6-13.8)	10.5 (−1.1-23.4)	7.1 (3.3-11.0) ***
University	3.6 (0.1-7.1)*	21.2 (12.7-30.2) ***	25.6 (12.2-40.5) ***	16.8 (12.4-21.4) ***
+ Physical exercise				
High school	1.1 (−2.0-4.3)	3.5 (−3.1-10.6)	4.7 (−5.7-16.3)	5.0 (1.6-8.6) **
University	3.3 (−0.2-6.9)	16.0 (8.0-24.6) ***	15.6 (3.8-28.9) **	13.1 (9.0-17.2) ***
+ Body mass index				
High school	1.2 (−1.9-4.4)	5.1 (−1.8-12.5)	6.9 (−4.3-19.5)	5.8 (2.2-9.6) **
University	3.6 (0.2-7.2)*	18.8 (10.4-27.9) ***	20.7 (7.6-35.4) **	15.0 (10.6-19.6) ***
+ Smoking				
High school	1.5 (−1.6-4.8)	6.9 (−0.3-14.6)	10.1 (−1.9-23.4)	7.4 (3.5-11.5) ***
University	4.1 (0.6-7.7)*	22.8 (14.1-32.3) ***	26.4 (12.4-42.2) ***	17.8 (13.2-22.7) ***
+ Alcohol				
High school	1.1 (−2.1-4.3)	3.8 (−3.2-11.4)	4.4 (−6.8-16.9)	4.9 (1.1-8.8) *
University	3.4 (−0.2-7.1)	18.2 (9.7-27.5) ***	17.5 (4.6-32.1) **	14.0 (9.5-18.6) ***
+ Chronic diseases and all lifestyle-related factors				
High school	0.5 (−2.6-3.6)	1.5 (−5.0-8.4)	1.9 (−8.0-12.7)	3.1 (−0.1-6.4)
University	2.2 (−1.2-5.8)	11.5 (3.8-19.8) **	9.8 (−1.2-22.0)	9.5 (5.7-13.4) ***

All models were adjusted for age and gender. In the other models, additional adjustments were made for chronic diseases, and each of the lifestyle-related factors, respectively. In the final model, age, gender, chronic diseases and all lifestyle-related factors were analysed together.

* indicates p < 0.05; ** indicates p < 0.01; and *** indicates p < 0.001.

**Figure 1:**
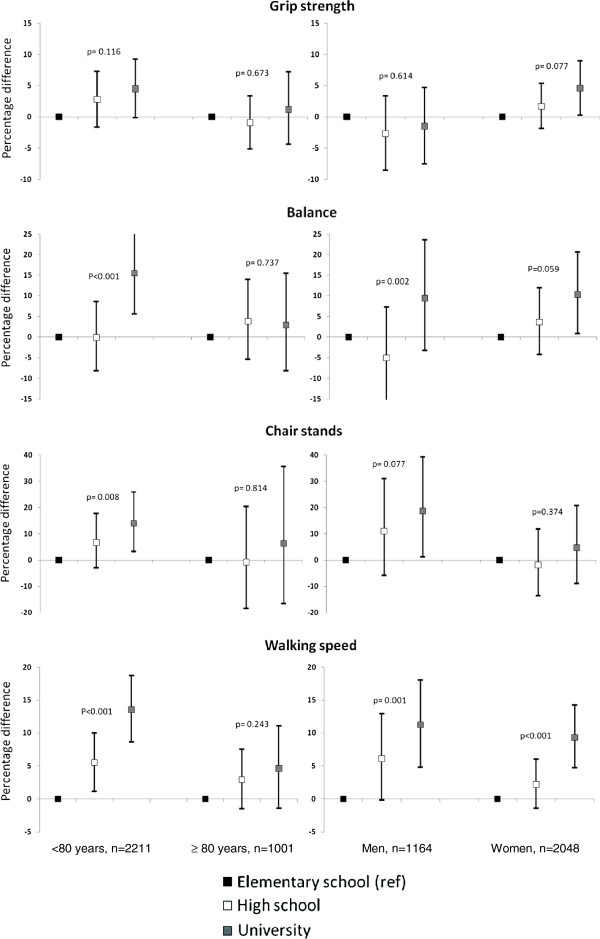
**Education-related differences and overall p-values for the association of education with grip strength, balance, chair stands and walking speed in people by age (<80 and ≥80 years) and gender.** The analyses were adjusted for age, gender (when not stratified by gender), chronic diseases and all lifestyle-related factors.

Age-stratified analyses revealed that education-related differences in grip strength were only found in people aged <80 years, but they were found for balance, chair stands and walking speed in people of all ages, in age and gender adjusted models (data not shown). In the fully adjusted model, the education-related difference in grip strength among people <80 years was attenuated and was no longer statistically significant (p _interaction_ of age group and education on grip strength = 0.216) (Figure 1). The differences in balance, chair stands and walking speed, including the gradient difference between levels of education and walking speed, remained statistically significant for people <80 years, but not for people aged 80+ years (p _interaction_ of age group and education on balance = 0.018, on chair stands = 0.896 and on walking speed = 0.056) (Figure 1).

Gender-stratified analyses revealed education-related difference in all four dimensions of physical performance among women, and in balance, chair stands and walking speed among men when adjusting for age (data not shown). In the fully adjusted model, women with university education had statistically significant better grip strength, balance and walking speed compared to women with elementary school education and men with university education had statistically significant better chair stands and walking speed compared to men with elementary school education (Figure 1). However, we did not detect any statistically significant interaction between education and gender (p _interaction_ of gender and education on grip strength = 0.896, on balance = 0.320, on chair stands = 0.516 and on walking speed = 0.482).

**Figure 2:**
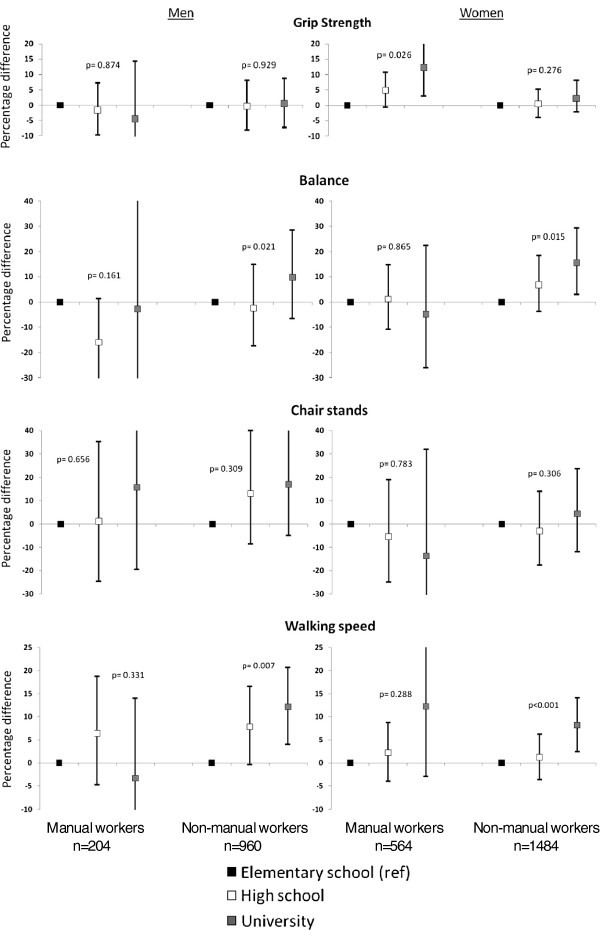
**Education-related differences and overall p-values for the association of education with grip strength, balance, chair stands and walking speed in men and women, respectively, stratified by occupational class (manual/non-manual workers).** The analyses were adjusted for age, chronic diseases and all lifestyle-related factors.

To investigate whether these associations are different in different occupational categories in men and women, stratified analyses of manual workers versus non-manual workers were performed in men and women, respectively. The results suggested that the education-related difference in grip strength was only evident among female manual workers, (p for the 3-way overall interaction between education, occupational class and gender on grip strength < 0.001). The difference in balance was only evident among female non-manual workers (p for the 3-way overall interaction between education, occupational class and gender on balance = 0.006). The differences in walking speed were significant among non-manual workers of both genders. However, an educational gradient was also observed among female manual workers (p for the 3-way overall interaction between education, occupational class and gender on walking speed < 0.001) (Figure 2). Non-significant associations were seen between education and chair stands among manual and non-manual workers among both genders (p for the 3-way overall interaction between education, occupational class and gender on chair stands = 0.086).

Finally, sensitivity analysis was performed in which subjects with imputed values were excluded from the analytical sample (n = 553 for grip strength, n = 189 for balance, n = 103 for chair stands and n = 136 for walking speed), and similar results were obtained.

## Discussion

In this population-based sample of older adults, we found education-related differences in balance and walking speed, even after adjustment for chronic diseases and lifestyle-related factors. However, the differences in grip strength and chair stands were attenuated by this adjustment. Analyses stratified by age group revealed that the differences were less evident in advanced age (80+ years). Gender-stratified analyses revealed that women with university education had significantly better grip strength, balance and walking speed compared to women with elementary school education and men with university education had significantly better chair stands and walking speed compared to men with elementary school education, in the fully adjusted models. Further analyses showed that the difference in grip strength was only evident in female manual workers, while the difference in balance and walking speed was only evident among female and male non-manual workers, respectively.

Earlier reports on age variations in social inequalities in health are contradictory [8-12]. Variations between measures of health used in the different studies have been suggested to contribute to the inconsistent results [12]. To our knowledge, this is the first study showing age variations in education-related differences in objectively measured physical performance. The education-related differences were smaller in people 80+ years compared to people <80 years in all four dimensions of physical performance in the fully adjusted model. This finding may have several possible explanations. First, exposure to psychosocial risk factors among persons with lower education or the impact of these factors on health may be greater among people in middle age and early old age compared to the oldest-old [8]. Secondly, the relative importance of education on physical performance may be less at older ages due to frailty [35]. Thirdly, the age differences may also reflect differences between birth cohorts. Finally, this finding could be explained by differential survival effects, since the oldest persons with worst physical function and lower education may be less likely to be included in the study due to death. However, this hypothesis was not supported by a study examining mortality selection in old age [11]. Since the interactions between education and age group were only significant for balance, borderline significant for walking speed and non-significant for grip strength and chair stands, no ultimate conclusions can however be drawn on the age differences.

We found that women with university education had significantly better grip strength, balance and walking speed compared to women with elementary school education and men with university education had significantly better chair stands and walking speed compared to men with elementary school education. Our results suggest that there are different patterns of education-related differences in physical performance in older men and women. The gender differences may be explained by differences in the types and durations of exposures in men and women with various levels of education, respectively, such as work-related exposures. However, since the interactions between education and gender were found to lack statistical significance for all outcomes, the results on the gender differences have to be interpreted with caution.

Stratified analysis by gender and occupational class showed that the education-related difference in grip strength was only evident among female manual workers. This may be explained by the fact that heavy manual work may maintain physical capacity, specific to the task that had been performed during work, in aging [36]. However, this pattern was not seen in men, perhaps because of differences in types and amounts of work-related tasks in male versus female manual workers. Previous reports on social gradients in grip strength are contradictory. One study found low income but not low education to be associated with poor grip strength [5]. Manual work has been associated with worse grip strength among people aged 80+ years [37], while others have reported no such association among younger populations [4]. The contradictory findings in the literature may be explained by differences between the cohorts, such as differences in age, countries, and education of the study populations [4,37]. Another explanation may be the modifying effect of occupational class on education-related differences in grip strength among women, as found in the present study.

Further stratified analysis by occupational class suggested that the education-related differences in balance in women and in walking speed in men were only evident among non-manual workers. One possible explanation may be that strenuousness of manual work contributes to poor performance in balance and walking speed independent of educational level. In addition, non-manual workers with higher education may be exposed to more favourable both work-related and non-work related factors compared with lower educated non-manual workers. However, it must be pointed out that since manual workers were fewer than non-manual workers, the lack of significant education-related differences among the manual workers in balance and walking speed may partly be explained by low statistical power. For the same reason, it is however less likely that the lack of education-related differences among female non-manual workers in grip strength is due to low statistical power.

Chronic diseases explained only a small part of the differences in physical performance in our findings. By contrast, a previous study reported that chronic diseases explained almost 40% of the education-related differences in mobility [6]. A possible explanation for the contradictory results may be the differences in the type of chronic diseases that were included. The previous study included a pre-selected list of common chronic diseases, while we included all prevalent chronic diseases. Moreover, other studies investigating the mediating effect of lifestyle on education-related differences in physical performance showed conflicting results [5-7,21]. We found that physical activity explained some of the education-related differences in physical performance, suggesting that promoting physical exercise may help to reduce some of the education-related differences in physical performance in old age. Since people in more socially disadvantaged groups may participate less frequently in leisure-time exercise [20], promoting participation in these groups may be an important public health target. However, the differences between results from various statistical models were not statistically significant for any of the outcomes.

A major strength of this study is the large sample of people, living both at home and in institutions, providing a complete picture of the general older population in our geographic location. Moreover, we employed objective testing of physical performance instead of subjective self-reported measures, and we used different sources of medical diagnoses, including direct clinical examination, thus limiting potential biases. However, the results for walking speed may have been biased by the different distances covered. The longer walk may better reflect a person’s normal walking speed than the shorter walk. However, it was not feasible to perform the longer walk at home visits due to restricted space. Excluding this group would have led to greater bias in the results, since the subjects assessed on home visits constitute a more disabled group in comparison with the entire sample. In addition, data from the National Health and Nutrition Survey (NHANES) showed that walking speed measured over the distances 2.4 and 6 meters are comparable [38]. Moreover, tests for walking speed are generally considered highly reliable, regardless of the distance [29,39,40].

Another limitation is the missing data on physical performance, especially on grip strength, which was missing for all subjects, assessed at home visits. We chose to impute the missing data on physical performance. Imputation of large amounts of missing data may lead to biased results, therefore the results for grip strength should be considered with caution. However, excluding this group probably would have led to greater bias in the results since the subjects with imputed data had lower education and worse physical performance compared with the entire sample. Other limitations of the study include the cross-sectional design, which restricts the ability to determine the direction of the observed associations. However, educational attainment was achieved long before the assessment of the outcomes. It is unlikely that directionality is questionable. Moreover, possible selective survival bias due to the cross-sectional design may have led to underestimations of the education-related differences, since persons with low education who also had worse physical performance may die earlier than highly educated persons [41]. Finally, health inequalities in Sweden, a country with a large nationalized system of public services, such as education, health care and social benefits, and extensive welfare [42], may be smaller than in some other countries.

## Conclusions

In this population-based study of people aged 60 years and older living at homes and in institutions, higher levels of education were associated with better balance, chair stands and walking speed in people younger than 80. However, the differences were less evident after the age of 80 years. Higher education was associated with better grip strength among female manual workers and better balance and walking speed among female and male non-manual workers, respectively.

## Abbreviations

SNAC: The Swedish National Study on Aging and Care; SNAC-K: The Swedish National Study on Aging and Care in Kungsholmen population study; BMI: Body mass index.

## Competing interests

The authors declare that they have no competing interest.

## Author contributions

Study concept and design: AKW, IK and HXW; Data analysis: AKW and IK; Interpretation of data and preparation of manuscript: AKW, IK, ER, SA and HXW. All authors have read and approved the final version of the manuscript.

## Pre-publication history

The pre-publication history for this paper can be accessed here:

http://www.biomedcentral.com/1471-2458/13/641/prepub
